# First molecular confirmations of *Anopheles dirus* and *Anopheles scanloni* in Indonesia, with DNA of zoonotic, enzootic and human malarias detected in *An. dirus*

**DOI:** 10.1038/s41598-026-42478-z

**Published:** 2026-03-02

**Authors:** Boni F. Sebayang, Bram van de Straat, Ahadi Kurniawan, Adzkia M. Haq, Triwibowo A. Garjito, Manop Saeung, Sylvie Manguin, Inke N. D. Lubis, Matthew J. Grigg, Tanya L. Russell, Thomas R. Burkot

**Affiliations:** 1 Australian Institute of Tropical Health and Medicine, James Cook University, Cairns, Australia; 2 Medan Public Health Laboratory Center, Ministry of Health of the Republic of Indonesia, Medan, Indonesia; 3 Faculty of Medicine, Universitas Sumatera Utara, Medan, Indonesia; 4 Vector-borne and Zoonotic Research Group, Research Center of Public Health and Nutrition, Health Research Organization, BRIN, Salatiga, Indonesia; 5 Department of Medical Entomology, Faculty of Tropical Medicine, Mahidol University, Bangkok, Thailand; 6 HSM, Univ. Montpellier, CNRS, IRD, Montpellier, France; 7 Menzies School of Health Research, Charles Darwin University, Darwin, Australia; 8 Institute for Health Research, University of Notre Dame Australia, Fremantle, Australia

**Keywords:** *Plasmodium knowlesi*, Leucosphyrus Group, *Anopheles dirus*, *Anopheles scanloni*, North Sumatra, Enzootic malaria, Diseases, Ecology, Ecology, Genetics, Microbiology, Molecular biology, Zoology

## Abstract

Despite Indonesia reporting high numbers of *Plasmodium knowlesi* malaria cases in North Sumatra Province, the *Anopheles* mosquito vectors remain unknown. This study identified the Leucosphyrus Group species present in Langkat Regency, North Sumatra, and the *Plasmodium* species DNA in their heads and thoraces. Mosquitoes collected by human landing catch were morphologically identified and their species identification subsequently confirmed using ITS2 sequencing as well as Dirus Complex (DiCSIP) and *Anopheles scanloni*-specific PCR. Reverse-transcription real-time and nested PCR assays targeting the 18 S rRNA gene were applied for *Plasmodium* species detection and identification. Of 597 morphologically identified Leucosphyrus Group mosquitoes, two species of the Dirus Complex were confirmed for the first time in Indonesia: 97.8% of specimens were *Anopheles dirus* with 2.2% being *Anopheles scanloni.* Seven *An. dirus* specimens were *Plasmodium*-positive, including mixed infections with *P. inui*,* P. knowlesi*, and/or *P. vivax* and one equivocal sample positive for *P. coatneyi* and *P. knowlesi.* BLAST analysis indicated possible cross-reactivity of *P. fieldi* primers with *P. inui.* This study provides the first molecular confirmation of *An. dirus* and confirms the presence of *An. scanloni*, two species of the Dirus Complex in North Sumatra. In addition, it demonstrates the presence of both macaque and human *Plasmodium* species DNA in *An. dirus*, suggesting the potential role of this species in zoonotic and human malaria transmission in this Indonesian region.

**Supplementary Information:**

The online version contains supplementary material available at 10.1038/s41598-026-42478-z.

## Introduction

The incidence of the major human malarias, *Plasmodium falciparum* and *P. vivax*, and their associated morbidity and mortality has diminished significantly in recent decades, although progress has stalled with cases rising in several regions^1,2^. To date, 47 countries and one territory are certified as having eliminated malaria since 1962, including 21 countries since 2000^3,4^. However, *Plasmodium knowlesi* is an enzootic malaria parasite in macaque monkeys and a naturally occurring zoonotic parasite in humans since the 1960s^5^. *Plasmodium knowlesi* has subsequently emerged as a significant zoonotic public health threat in Southeast Asia, where human infections are increasingly reported^4,6^. Although long-tailed (*Macaca fascicularis*) and pig-tailed macaques (*Macaca nemestrina* and *Macaca leonina*) are the major reservoir hosts, other primate species, such as *Presbytis* spp., can also harbour *P. knowlesi*^7^. In Indonesia, the first human case of *P. knowlesi* was documented in South Kalimantan in 2010^8^. Since then, cases have been reported in the provinces of Kalimantan (Central, South and East Kalimantan) and Sumatra Islands (Aceh, Jambi, West Sumatra and North Sumatra)^8–15^. Despite 377 reported human *P. knowlesi* cases in North Sumatra in 2017, the vector(s) responsible remain unknown^15–17^.

There are eight confirmed *Anopheles* vectors of *P. knowlesi* in Southeast Asia, including five species from the Leucosphyrus Group: three from the Leucophyrus Complex (*Anopheles balabacensis*, *Anopheles latens*, and *Anopheles introlatus)*, and two species from the Dirus Complex (*Anopheles dirus* and *Anopheles cracens*)^18–22^, as well as one species from the Barbirostris Group and Complex (*Anopheles donaldi*^23,24^. Two species in the Umbrosus Group, *Anopheles collessi* and *Anopheles roperi* were morphologically identified as vectors of *P. knowlesi*; however, they could not be delineated phylogenetically using COI sequences^25,26^. *Anopheles hackeri* was reported as a vector of *P. knowlesi* and other enzootic malarias in the 1960s^27,28^, but there have been no subsequent reports using molecular incrimination methods. *Anopheles sundaicus* is a suspected *P. knowlesi* vector based on a finding of a single positive pool of whole mosquitoes in the Andaman and Nicobar Islands, India^29^. *Anopheles kochi* appears to be potential vector that might play a role in the transmission of *P. knowlesi* in Singapore^30^.

There are limited data on the distributions, behaviours, and vector competences of the Leucosphyrus Group members for zoonotic malaria in Southeast Asia^16^. North Sumatra, Indonesia, is an understudied region for investigating the *Anopheles* vectors of simian malaria^16,17^. On Sabang Island in neighbouring Aceh Province, *An. dirus s.l.* and *An. cracens* have been morphologically identified^31–33^. In the Langkat Regency, North Sumatra, Leucosphyrus Group mosquitoes were the most prevalent *Anopheles* mosquitoes collected^34^. Understanding the distribution and species composition and malaria vector status of the Leucosphyrus Group is critical for developing targeted control strategies for both zoonotic and human malarias, as even closely related *Anopheles* species may vary in feeding preferences, resting habits, and susceptibility to insecticides, which will impact the effectiveness of vector control strategies. This study analysed samples of morphologically identified Leucosphyrus Group mosquitoes from North Sumatra by PCR to molecularly confirm the mosquito species present. It also assessed their potential role as malaria vectors by molecular analyses for *Plasmodium* species in the head/thorax.

## Results

### Members of the Dirus Complex

**Fig. 1:**
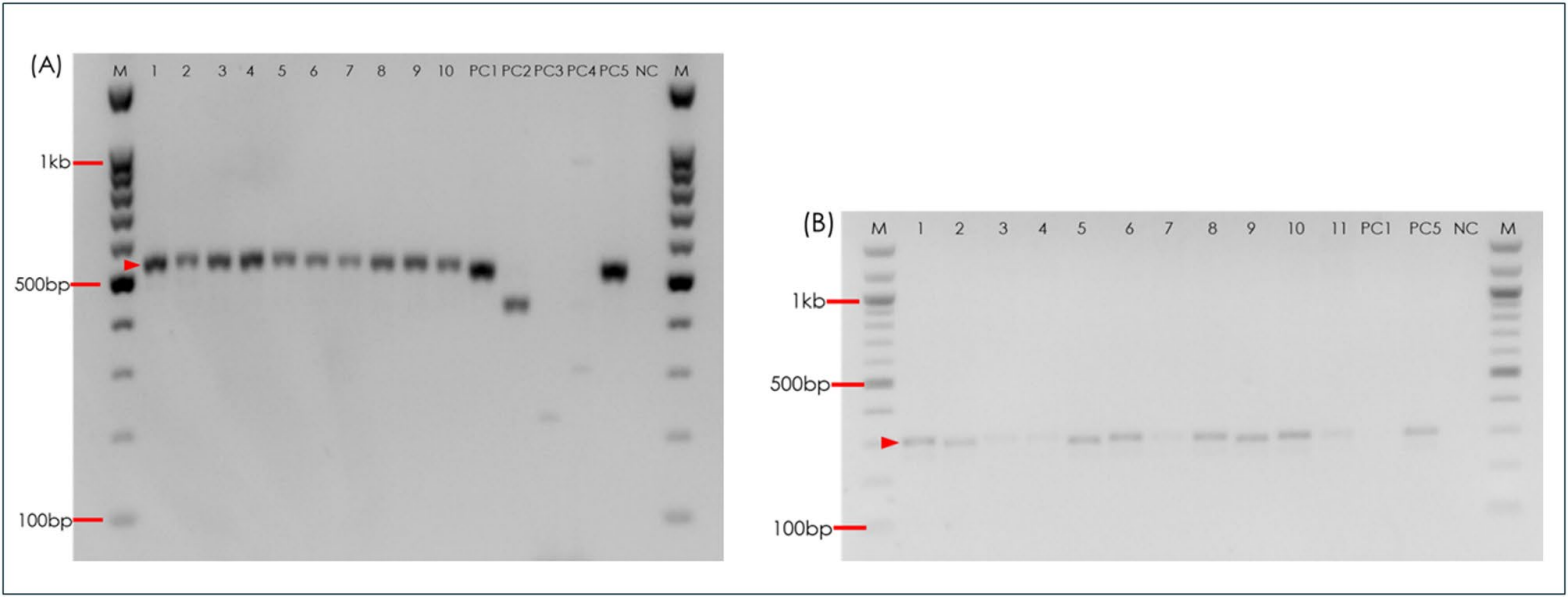
Amplification of *An. dirus* and *An. scanloni* by PCR assay. (**A**) ITS2 DiCSIP assay results. No1-10: collected specimens; PC1: *An. dirus* (521 bp); PC2: *An. cracens* (435 bp): PC3: *An. baimaii* (225 bp); PC4: *An. nemophilous* (301 bp); PC5: *An. scanloni* (528 bp); NC: Negative control; M: 100 bp marker. (**B**) ITS2 SSP assay results. No.1–10: collected specimens; PC1: *An. dirus* (no amplification product); PC5: *An. scanloni* (300 bp); NC: negative control; M: 100 bp marker.

Of 597 morphologically identified Leucosphyrus Group mosquitoes, two species were found: 584 *Anopheles dirus* specimens were (97.8%) identified by DiCSIP and 13 *Anopheles scanloni* (2.2%) by the SSP assay (Fig. 1). The initial ITS-PCR assay could not distinguish the specific species within the Leucosphyrus Group. The DNA band sizes of the specimens were similar to those of the positive controls, *An. balabacensis* from the Leucosphyrus Complex and *An. cracens* from the Dirus Complex (MW = mean ± SE, 873 ± 1.39) (Supplementary Fig. S1). Representative Leucosphyrus Group specimens from each dusun were confirmed as *An*. *dirus* based on ITS2 sequence analysis, with BLAST alignment showing ≥ 99.9% identity to reference sequences. Nine high-quality ITS2 sequences (729 bp) with clear chromatograms were generated and deposited in GenBank under accession numbers PQ589932–PQ589940.

*Anopheles dirus* was collected in both Dusun II (61) and Dusun V (523), Ujung Bandar Village, Salapian Subdistrict, Langkat Regency, North Sumatra. Interestingly, *An. scanloni* was only identified in Dusun V.

### *Plasmodium* genus and species identification

Thirteen *An. dirus* specimens (2.2%; 95% CI: 1.2–3.8%) were positive (Ct value < 40) by *Plasmodium* genus RT-qPCR^35^ and proceeded to definitive species-specific testing by RT-nPCR and RT-hemi nPCR^7,36,37^(Table 1; Supplementary Table S1). These included 6 samples with equivocal Ct values of between 35 and 40, which remained positive on repeat testing at 1:10 cDNA dilution to minimise confounding by potential PCR inhibitors. *Plasmodium* cDNA was not detected in any *An. scanloni* specimens.

**Table 1:** Identification of zoonotic, enzootic and human *Plasmodium* species in *An. dirus*.

No.	Plasmodium genus identification^1^		Zoonotic malaria^2^				Enzootic malaria^3^	Human malaria^4^	
	Ct value x̄ (± SD)	Results	Pin	Pcy	Pct	Pfiel	Pk	Pv	Pf, Pm, Po
1	24.7 (± 0.1)	Positive	+	-	-	+/-	-	+	-
2	25.5 (± 0.4)	Positive	+	-	-	+/-	-	+	-
3	27.8 (± 0.1)	Positive	+	-	-	+/-	-	+	-
4	28.0 (± 0.4)	Positive	+	-	-	-	+	-	-
5	30.1 (± 0.8)	Positive	+	-	-	+/-	+	+	-
6	33.0 (± 0.5)	Positive	-	-	-	-	-	-	-
7	34.2 (± 0.4)	Positive	-	-	-	-	-	-	-
8	35.1 (± 0.4)	Positive	-	-	-	-	-	-	-
9	35.2 (± 0.9)	Positive	-	-	-	-	-	-	-
10	36.2 (± 1.8)	Positive	-	-	-	-	-	-	-
11	37.3 (± 1.4)	Positive	-	-	-	-	-	-	-
12	38.4 (± 0.7)	Positive	-	-	-	-	-	-	-
13	39.2 (± 0.5)	Positive	-	-	+	-	+	-	-

^1^RT-qPCR assay by Kamau et al., 2011.

^2^RT-nPCR assay by Lee et al., 2011.

^3^RT-hemi nPCR PCR assay by Imwong et al., 2009.

^4^RT-nPCR assay by Snounou et al., 1993.

Pf: *Plasmodium falciparum*; Pv: *P. vivax*; Pm: *P. malariae*; Po: *P. ovale*; Pk: *P. knowlesi*;

Pin: *P. inui*; Pcy: *P. cynomologi*; P. ct: *P. coatneyi*; Pfiel: *P. fieldi*. +: positive result; -: negative resul.

**Fig. 2:**
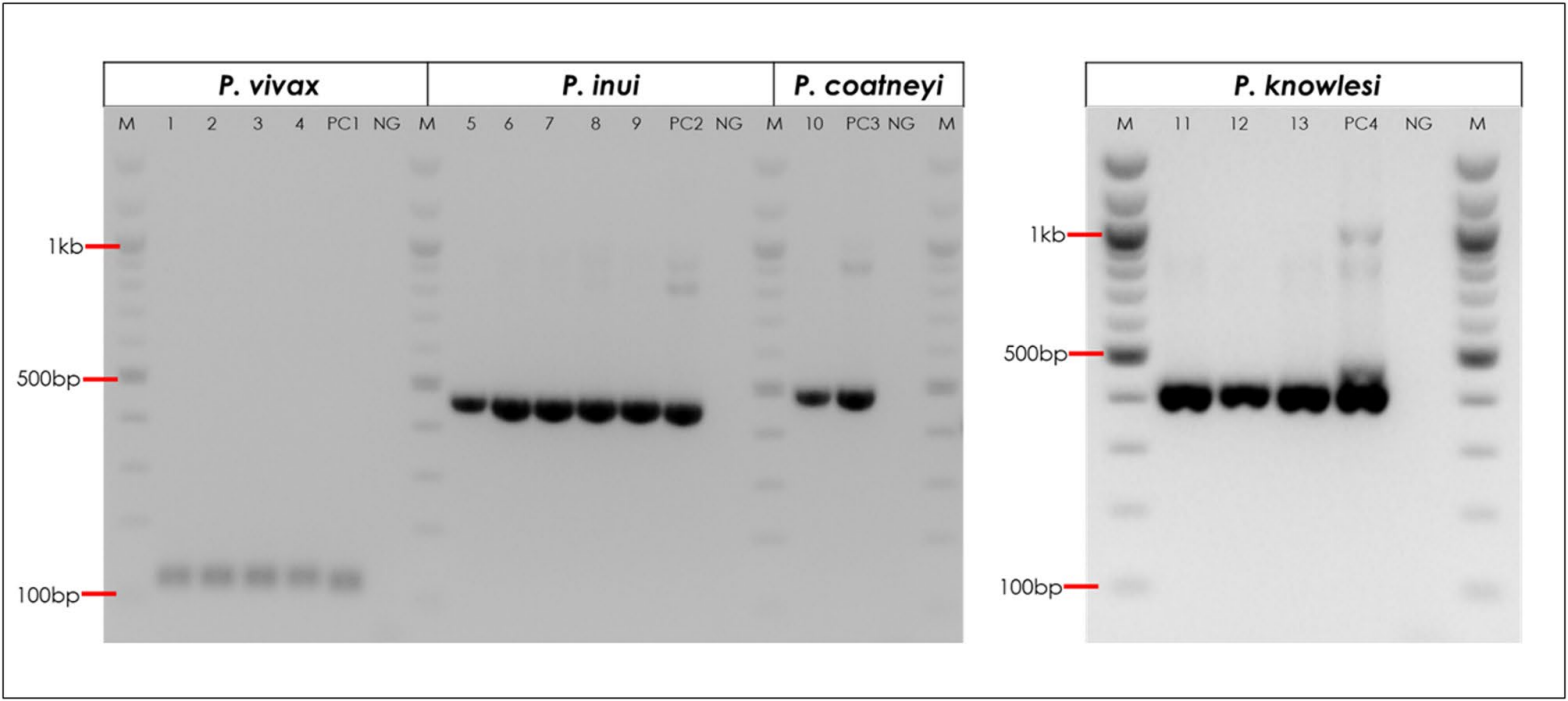
Positive *Plasmodium* species from RT-nPCR results. Lanes 1–4: positive *P. vivax* (120 bp); Lanes 5–9: positive *P. inui* (479 bp); Lane 10: positive *P. coatneyi* (503 bp); Lane 11–13: positive *P. knowlesi* (410 bp). PCs: positive controls; NG: negative control: M: 100 bp ladder marker. The 1.5% agarose gel (EtBr 10 mg/ml) was run at 100 volts for 85 min.

Six of 584 (1.0%; 95% CI: 0.4–2.2%) *An. dirus* specimens had *Plasmodium* species infections confirmed on nested species-specific assays, all were mixed species co-infections (Table 1; Fig. 2). The *P. knowlesi* infection prevalence in *An. dirus* was 0.5% (3/584; 95% CI 0.1–1.5%). The three *P. knowlesi-*positive *An. dirus* cDNA specimens were co-infected: one with *P. inui*, *P. fieldi* and *P. vivax*, one with *P. inui* alone, and one with *P. coatneyi*, respectively. *Plasmodium inui* was the most common macaque *Plasmodium* species found in *An. dirus*, with five positive specimens (0.6%; 95% CI: 0.4-3.0%), including four being also positive for both human malaria, *P. vivax*, and zoonotic *P. fieldi*. Specimens were not positive for *P. cynomolgi* or other human species *P. falciparum*, *P. malariae* or *P. ovale* spp.

Species-specific PCR products were sequenced by the Sanger method and compared with available GenBank database references for *P. vivax*, *P. inui*, *P. coatneyi*,* P. fieldi*, and *P. knowlesi*. The majority of BLAST results matched the RT-nPCR results, except for RT-qPCR positives for *P. fieldi*, which were identified as *P. inui* by BLAST.

## Discussion

With a population of at least 15.3 million people in 2023^38^, over 50% of whom live in rural areas, understanding the at-risk population for zoonotic malaria in North Sumatra, Indonesia, depends on the identification and distribution of the known vector species. Understanding and responding to the emerging threat of zoonotic malaria in Southeast Asia requires detailed comprehensive knowledge of vector species distributions, their ecological preferences, and the behaviours influencing their interactions with macaques and humans and the *Plasmodium* parasites that they harbour^16,39^. This study addressed major knowledge gaps on the mosquito species and *Plasmodium* species prevalence of vectors of zoonotic, enzootic and human malarias in Langkat Regency, North Sumatra, Indonesia. We confirmed that *An. dirus* is the predominant member of the Leucosphyrus Group in the study area and represents a potentially important vector of *P. knowlesi*, with an observed infection prevalence of 0.5% (95% CI: 0.1–1.5%). Co-infections and the transmission potential of other known but rare zoonotic malarias, such as *P. inui* and *P. coatneyi* in *An. dirus*, were identified. In addition, the known range of *An. scanloni* was broadened by this study to include the Indonesian island of Sumatra. However, the small numbers of *An. scanloni* found coupled with the absence of *Plasmodium* species detections in this mosquito suggest it may not be a major malaria vector in Sumatra.

The spatial distribution of species within the Leucosphyrus Group remains poorly defined across South and Southeast Asia, including the large province of North Sumatra. Because of potential shifts in distributions, occurrence mapping of the major zoonotic parasite *P. knowlesi* requires ongoing surveillance to understand and refine changing transmission risk estimates^40–43^. Our study contributes to this effort by documenting the presence of *An. dirus* in North Sumatra, broadening the recognized distribution of this species significantly beyond the Greater Mekong Subregion. As *An. dirus* is a well-established vector of *P. knowlesi* and human malaria in mainland Southeast Asia^16^, its detection in this area raises important considerations for zoonotic and human malaria transmission risk in Indonesia.

*Anopheles dirus* and *An. scanloni* both belong to the Dirus Complex within the Leucosphyrus Group (*Cellia*) in the Neomyzomyia Series^41,44^. The Dirus Complex consists of three additional known species found in *P. knowlesi* enzootic areas: *An. cracens*, *An. baimaii*, and *An. nemophilous* (all found in mainland Southeast Asia in sympatric areas for long- and pig-tailed macaque hosts); as well as two species not found in *P. knowlesi* enzootic areas, *An. elegans* (found in Sri Lanka and southwestern India), and *An. takasagoensis* (in Taiwan)^41,45–47^. In addition, *An.* aff. *takasagoensis*, a potential member of the Dirus Complex, refers to morphologically similar populations found in northern Vietnam that are genetically distinct from *An. takasagoensis* from Taiwan^48^.

Species-specific identifications within the Dirus Complex are challenging due to overlapping morphological characteristics among the sibling members^41,45^. Challenges in distinguishing sibling species based on morphology alone are well documented across several *Anopheles* species complexes, including *Anopheles culicifacies*, where cryptic sibling species differ in vectorial roles and epidemiological importance, necessitating the use of molecular or complementary identification approaches^49,50^. *Anopheles dirus*, previously known as *An. dirus* A, has been identified morphologically and/or molecularly in Thailand, Myanmar, Cambodia, Lao PDR, Vietnam, and southern China (Hainan Island)^16,51–55^. In Indonesia, *An. cracens* (former *An. dirus* B) has only been reported based on morphology and only in Sabang Island (Aceh), Sumatra^31,41,56^. In addition, *An. scanloni* (former *An. dirus* C) had a known distribution along the Myanmar-Thailand border^57,58^, but has for the first time now been reported in Indonesia. Until recently, species differentiation relied primarily on allele-specific multiplex PCR^59^. However, this method often produced nonspecific and inefficient amplification, raising uncertainty as to species identification accuracy^60^. Hence, *An. dirus s.l.* previously reported in some areas may include both *An. dirus* and *An. scanloni* in sympatry. The recent development of species-specific PCRs for *An. dirus* (DiCSIP) and *An. scanloni* (SSP)^60^ and their use in the current study has strengthened the validity and improved the reliability of distinguishing members of the Dirus Complex, including *An. dirus* and *An. scanloni*.

The presence of *An. dirus* and *An. scanloni* in North Sumatra were further confirmed in this study by the use of targeted ITS2 gene sequencing. However, sequence data is resource-intensive, requiring specialised laboratory equipment, skilled personnel, and substantial computational resources, and its accuracy is highly dependent on the availability of a comprehensive and well-curated reference database. The absence of complete ITS2 sequence data for *An. scanloni* and *An. nemophilous* creates uncertainty in identifying the members of the Dirus Complex^60^ by targeted ITS2 gene sequencing.

*Anopheles dirus* is an important enzootic, zoonotic and human malaria vector species. Sporozoites of *P. falciparum* and *P. vivax* were detected by CSP-ELISA and PCR (18 S rRNA) in *An. dirus* in Thailand^61–66^, Cambodia^67^, Lao PDR^68,69^, Vietnam^70^, southern China (Hainan Island)^71^, and in *An. baimaii* Myanmar^72,73^ and India^74^. *Anopheles dirus* and *An. cracens* were confirmed as vectors of *P. knowlesi* using molecular assays including PCR targeting the parasite CSP and 18 S rRNA genes, RT-PCR for CSP mRNA and sporozoite surface protein 2 (SSP2) mRNA and Sanger sequencing based on the CSP gene^75–77^. In previous studies, both *An. dirus* and *An. cracens* salivary glands and human blood samples were found to be positive both for *P. knowlesi* and co-infected with *P. falciparum* and/or *P. vivax*^75–77^. In this study in North Sumatra, evidence of infections (e.g., DNA) of the zoonotic malaria, *P. knowlesi*, in *An. dirus* were found with an estimated prevalence of 0.5%. This prevalence is lower than reported for *An. balabacensis* in Sabah (Malaysia), where infection rates of *P. knowlesi* in 2015 were 0.9%^20^. Importantly, while human cases of *P. knowlesi* have been sporadically reported in Sumatra, the detection of infections in *An. dirus* provides the first entomological evidence of a potential vector in this region, underscoring a moderate but significant zoonotic transmission risk.

In laboratory experiments, *An. dirus s.l.* supported *P. cynomolgi*, *P. inui*, *P. fieldi* and *P. coatneyi* development to the sporozoite stage^78–81^. The transmission of additional natural macaque *Plasmodium* species in SEA has yet to be confirmed in *An. dirus* populations^33^. In this study, total nucleic acids were extracted, and RNA was reverse transcribed into cDNA, allowing highly sensitive detection of the enzootic malarias, *P. inui* and *P. coatneyi*, in *An. dirus*. This approach also revealed potential *P. vivax* co-infections, echoing findings from Vietnam where co-infections of *P. falciparum*,* P. vivax* and *P. knowlesi* were reported^75,77^.

Macaque malaria parasite co-infections have been commonly reported^75,77^, (including in this study in North Sumatra), and diagnostic challenges remain regarding the specificity of many of the primers used to detect 18S ssu rRNA or other common gene targets for these highly genetically related *Plasmodium* species^37,82^. *Anopheles balabacensis*, a member of the Leucosphyrus Complex, was reported in Sabah and Sarawak, Malaysia, to be co-infected with combinations of *P. knowlesi*, *P. cynomolgi*, *P. fieldi*, *P. inui*, and *P. vivax* as detected by PCR^23,83^. *Plasmodium knowlesi* is predominantly a mono-infection in humans^84^. It is possible that some of these co-infections may reflect, either a degree of primer cross-reactivity among closely genetically related *Plasmodium* species in macaque hosts, or limitations in the number, diversity and annotations of GenBank reference sequences used for comparative species analyses, as was suggested in this study by *P. fieldi* specimens identified by PCR later identified as *P. inui* by BLAST. Furthermore, primer cross-reactivity between *P. cynomolgi* and *P. vivax* has also been reported^82,84^. However, it must be noted that other studies failed to find *P. cynomolgi* primer amplification and the robust design and validation of the separate *P. knowlesi* hemi-nested assay for specificity against other macaque *Plasmodium* species^37^ support the major infection prevalence findings in this study.

In this study, 13 *An. dirus* specimens tested positive for the *Plasmodium* genus, with six specimens identified to *Plasmodium* species. Several factors may explain these discrepancies. Firstly, the RT-qPCR assay to detect the genus *Plasmodium* has a lower limit of detection for at least *P. knowlesi* and *P. cynomolgi* compared to the species-specific nested PCR assays^35,85^. Secondly, the species-specific identification assays used in this study were designed primarily for detecting human and macaque *Plasmodium* species^7,35,37^. This raises the small possibility that species of the Dirus Complex, particularly *An. dirus* and *An. cracens* may harbor enzootic malaria parasites from other hosts.

Most mosquito specimens with borderline Ct values (in the range of 35–40) in the *Plasmodium* genus screening qPCR were failed to yield species-level identifications. These results were considered inconclusive, possibly as a consequence of either very low parasite density infections or due to false positives arising from technical artefacts such as non-specific amplification, degraded or low-concentration DNA, cross-contamination, or amplification near the limit of detection of the assay^86–88^. Importantly, high Ct values may indicate a low quantity of *Plasmodium* DNA in *Anopheles* vectors, potentially reflecting low parasitemias in vectors (and humans), which is often observed in the early stages of zoonotic malaria infections^89^. However, in this study, one sample with a borderline Ct value (39.2 ± 0.5) was confirmed as a mixed infection with *P. knowlesi* and *P. coatneyi*. This finding underscores the importance of maximising the sensitivity of genus-level screening assays, followed by species-specific analyses for low-level positives (with high/borderline Ct values), particularly in the context of zoonotic malaria surveillance.

While *An. dirus* is typically known for its anthropophilic behaviour, reports have also shown that *An. cracens* is an anthropophilic species^58^. However, variations in host feeding preferences observed in concurrent studies (unpublished data), along with reports of less anthropophilic tendencies in certain locations, suggest that *An. dirus’* role as a malaria vector may vary depending on ecological factors^52^. Little is currently known about the bionomics and ecological preferences of *An. dirus* in Indonesia (Sumatra), an important knowledge gap for assessing its local role as a vector. Addressing these gaps, particularly in host-seeking behaviours, biting times, and ecological drivers of its distribution, will be crucial for refining zoonotic risk estimates and developing integrated surveillance strategies.

Although *An. scanloni* was not positive for *Plasmodium* DNA in this study, *An. scanloni* is known as a secondary vector of *P. falciparum* and *P. vivax* in Thailand^53,90^ suggesting that its involvement in malaria transmission in North Sumatra cannot be excluded. The absence of *Plasmodium* infections in the *An. scanloni* analysed here may be due to the limited sample size in this study rather than a lack of vector potential. Future entomological investigations incorporating larger sample sizes will be crucial to elucidate the vector status and vectorial capacity of *An. dirus* and *An. scanloni* in this region.

## Conclusion

Two species of the Dirus Complex, *An. dirus* and *An*. *scanloni* were identified in North Sumatra, Indonesia. *Anopheles dirus* harboured the DNA of both macaque and human malaria parasites, including *P. knowlesi*, *P. inui*, *P. coatneyi*, and *P. vivax*. *Plasmodium* DNA was not detected in *An. scanloni*. These findings suggest that *An. dirus* may play an important role as a vector of zoonotic, enzootic and human malarias in North Sumatra, justifying intensified surveillance to better understand both its distribution and its role in malaria transmission, and its amenability to public health control measures in North Sumatra.

## Methods

### Study site and mosquito collection

Sites for mosquito sampling were based on a *P. knowlesi* regional transmission predictive risk map^40,91^ using estimates of the distributions of Leucosphyrus Group mosquitoes, macaques and human population densities, and environmental land types present to classify high-risk health facility catchment areas. The study was carried out in two of nine hamlets (dusuns), ‘Dusun II’ (3.371720, 98.328547) and ‘Dusun V’ (3.341947, 98.335406) of Ujung Bandar Village, Salapian Subdistrict, Langkat Regency, North Sumatra, Indonesia, where malaria cases in humans (including *P. knowlesi*) were previously identified by human surveillance in 2022–2023 [I. N. D. Lubis et al., unpublished]. The village is located in a hilly area at an elevation of 300–600 m above sea level, surrounded by oil palm plantations, small-scale mixed agriculture areas, and patches of disturbed forest. The area receives over 3000 mm of rainfall annually, with daytime temperatures typically ranging from 29 to 34 °C and nighttime temperatures from 19 to 24 °C.

Mosquitoes analysed had been sampled by human landing catch (HLC)^92^ at six sampling stations per Dusuns (two collectors per station) from July 2022 to June 2023^34^. Sampling was conducted over four nights per month from 18:30 to 06:00, alternating between the two Dusuns. The HLC is considered the gold-standard method for collecting host-seeking female mosquitoes and is recommended by the WHO for assessing entomological surveillance indicators and evaluating the efficacy of vector control interventions^93,94^. Furthermore, the HLC was selected to sample anophelines as a Latin square study confirmed that other sampling methods were ineffective relative to the HLC^34^.

**Fig. 3:**
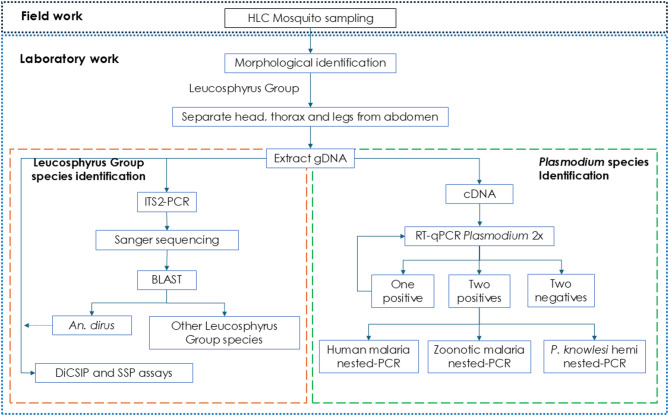
Workflow for mosquito collection, species identification of Dirus Complex mosquitoes and malaria species detection and identification.

Mosquitoes were morphologically identified in a field laboratory to genus, group or species, when possible, using taxonomy keys, including O’Connor and Soepanta (1989) for Indonesian *Anopheles* mosquitoes and Sallum et al. (2005) for members of the Leucosphyrus Group^41,95^. Adult mosquitoes were then placed individually in 1.5 mL Eppendorf tubes containing cotton wool and silica gel and transported to the laboratory at ambient temperature. All samples were stored at room temperature until molecularly analysed to identify mosquito and *Plasmodium* species (Fig. 3).

### DNA extraction and reverse transcription

The DNA of the heads and thoraces of individual adult Leucosphyrus Group mosquitoes were extracted separately using the DNeasy Blood & Tissue Kit (Qiagen, Hilden, Germany) according to the manufacturer’s protocol. A 10 µL aliquot of genomic DNA (gDNA) from each extracted sample was transformed into complementary DNA (cDNA) using a high-capacity cDNA reverse transcription PCR kit (Applied Biosystems by Thermo Fisher Scientific, Vilnius, Lithuania) for *Plasmodium* detection assays.

### Identification of species in the Leucosphyrus Group

All morphologically identified specimens of the Leucosphyrus Group were analysed for mosquito species identification using gDNA PCR amplification of the ribosomal internal transcribed spacer 2 (ITS2) region^60,96^. Initially, a single point ITS2 PCR using primers ITS2A and ITS2B confirmed which specimens belonged to the Leucosphyrus Group (Supplementary Table S2). The ITS2 amplification products of thirty Leucosphyrus Group specimens were subsequently sequenced at the Macrogen facility (Macrogen Inc., Seoul, Korea) and analysed through the Basic Local Alignment Search Tool (BLAST) in the NCBI database.

Specimens identified as belonging to the Dirus Complex from ITS2 sequences were then analysed using the molecular identification PCR (DiCSIP) and an *An. scanloni*‑specific PCR (SSP)^60^(Supplementary Table S2). Five positive controls of species in the Dirus Complex (i.e., *An. dirus*, *An. cracens*, *An. scanloni*, *An. baimaii*, and *An. nemophilous)* were provided by the Department of Entomology, Faculty of Agriculture, Kasetsart University (KU) and National Center for Genetic Engineering and Biotechnology (BIOTEC), Thailand. Negative controls (nuclease-free water without DNA template) were included in each PCR run to monitor for contamination and non-specific amplification. Detailed assay protocols for the Leucosphyrus Group species identifications are available in the Supplementary Methods.

### *Plasmodium* detection and identification

The *Plasmodium* genus 18S rRNA gene from the small ribosomal subunit was amplified from mosquito cDNA samples run in replicate using a real-time quantitative polymerase chain reaction (RT-qPCR) assay^35,85^(Supplementary Table S3). Samples generating discrepant replicant results, such as a > 3 difference in Ct values, or when one of the duplicates yielded an equivocal or negative result while the other duplicate was positive, were repeated. *P. knowlesi* DNA from a molecularly confirmed human clinical mono-infection served as the *Plasmodium* genus control for all samples. Positive results were defined as having a cycle threshold (Ct) value of < 40, consistent with human surveillance protocols. Samples with Ct values between 35 and 40 were considered potential positives and were retested using a 1:10 dilution of cDNA template to assess possible matrix-derived PCR inhibition. If Ct values remained < 40 upon retesting, the samples proceeded to species-specific *Plasmodium* assays for definitive identification. Negative results were defined as having a Ct value of > 40 in duplicate.

Positive and equivocal samples for the screening *Plasmodium* genus were then analysed using reverse transcriptase nested polymerase chain reaction (RT-nPCR) with the same gene target (18S rRNA) to detect both human (*P. falciparum*, *P. vivax*, *P. malariae* and *P. ovale*) and macaque malaria parasites (*P. knowlesi*,* P. inui*, *P. cynomolgi*, *P. coatneyi and P. fieldi*)^7,36,37^(Supplementary Table S3) with results visualised by 1.5% agarose gel electrophoresis. Clinical isolates for each human *Plasmodium* species and *P. knowlesi* were used as positive DNA controls, with validated gBlocks™ synthetic controls for other enzootic *Plasmodium* species such as *P. inui*, *P. cynomolgi*, *P. coatneyi and P. fieldi*^85^. Negative controls were included in every PCR reaction batch to verify the absence of cross-contamination. Detailed assay protocols for *Plasmodium* detection and identification are available in Supplementary Methods.

## Supplementary Information

Below is the link to the electronic supplementary material.

Supplementary Material 1

## Data Availability

The data sets used during this study are available in the JCU Research Data repository (10.25903/rabx-g630). The DNA sequence data generated in this study are available through the GenBank database (https://www.ncbi.nlm.nih.gov/genbank/). The ITS2 sequences of An. dirus from North Sumatra have been deposited under accession numbers PQ589932 - PQ589940.
