# Supplementary material for: First molecular confirmations of Anopheles dirus and Anopheles scanloni in Indonesia, with DNA of zoonotic, enzootic and human malarias detected in An. dirus
Source: Sci Rep. 2026 Mar 2;16:11572. doi: 10.1038/s41598-026-42478-z (PMC13057228; doi:10.1038/s41598-026-42478-z)
Supplement: Supplementary file 1 — Supplementary Material 1 [file 41598_2026_42478_MOESM1_ESM.docx]

**Supplementary information**


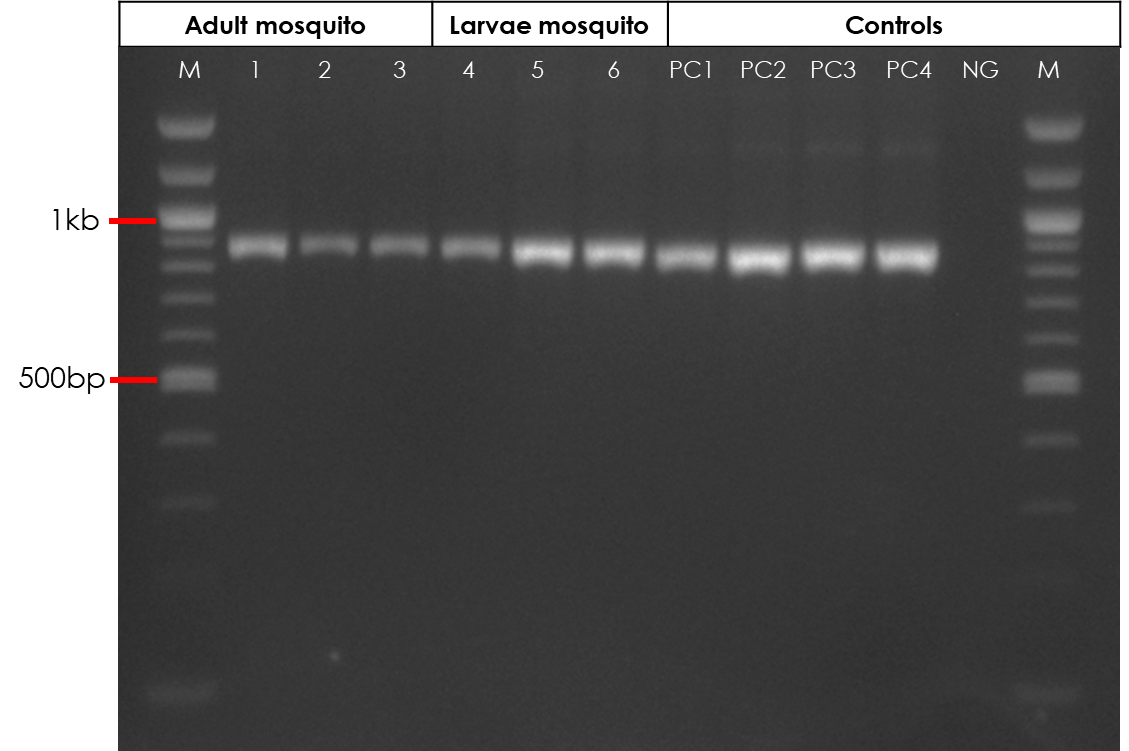


**Figure S1.** ITS2 PCR results of Leucosphyrus Group. All amplification products showed similar band sizes of members of the Leucosphyrus Group (MW = mean ± SE, 873 ± 1.39). Lanes 1-6: morphologically identified Leucosphyrus Group specimens; PC1 and PC2: *An. balabacensis* (Leucosphyrus Complex); PC3 and PC4: *An. cracens* (Dirus Complex); NG: negative control; M: 100-bp ladder marker

**Table S1.** Sampling location of *Anopheles* specimens positive for *Plasmodium* species

| **No.** | ***Anopheles* species** | **Dusun  (Hamlet)** | **Sampling  station** | **Latitude** | **Longitude** | **Land use type** | **Positive *Plasmodium* species identification** |
| --- | --- | --- | --- | --- | --- | --- | --- |
| 1 | An. dirus s.s | 5 | 4 | 3.341142 | 98.33489 | Mixed agriculture | *P. inui, P. vivax* |
| 2 | *An. dirus s.s* | 5 | 6 | 3.341359 | 98.33392 | Mixed agriculture | *P. inui, P. vivax* |
| 3 | *An. dirus s.s* | 5 | 4 | 3.341142 | 98.33489 | Mixed agriculture | *P. inui, P. vivax* |
| 4 | *An. dirus s.s* | 2 | 4 | 3.373406 | 98.32712 | Oil palm plantation | *P. inui, P. knowlesi* |
| 5 | *An. dirus s.s* | 5 | 4 | 3.341142 | 98.33489 | Mixed agriculture | *P. inui, P. knowlesi, P. vivax* |
| 6 | *An. dirus s.s* | 5 | 4 | 3.341142 | 98.33489 | Mixed agriculture | *Plasmodium spp.* |
| 7 | *An. dirus s.s* | 5 | 4 | 3.341142 | 98.33489 | Mixed agriculture | *Plasmodium spp.* |
| 8 | *An. dirus s.s* | 2 | 2 | 3.373835 | 98.32533 | Oil palm plantation | *Plasmodium spp.* |
| 9 | *An. dirus s.s* | 2 | 2 | 3.373835 | 98.32533 | Oil palm plantation | *Plasmodium spp.* |
| 10 | *An. dirus s.s* | 5 | 6 | 3.341359 | 98.33392 | Mixed agriculture | *Plasmodium spp.* |
| 11 | *An. dirus s.s* | 2 | 2 | 3.373835 | 98.32533 | Oil palm plantation | *Plasmodium spp.* |
| 12 | *An. dirus s.s* | 5 | 4 | 3.341142 | 98.33489 | Mixed agriculture | *Plasmodium spp.* |
| 13 | *An. dirus s.s* | 2 | 2 | 3.373835 | 98.32533 | Oil palm plantation | *P. coetneyi, P. knowlesi* |

**Supplementary Methods: Protocol for molecular identification of *Anopheles* and *Plasmodium* species**

1. **Identification of the Leucosphyrus Group members**
2. **Single-point ITS2 PCR for Sanger sequencing**

A single-point PCR targeted the ITS2 gene using ITS2A and ITS2B (Table S2), as described by Beebe and Saul (1995). The amplification master mix consisted of 1x GoTaq® flexi green buffer (Promega, Madison, WI, USA), 3.0 mM MgCl2 (Promega), 0.2 mM dNTP mix (Promega), 0.5 µM of each ITS2A and ITSB primers in a total volume of 30 µL (Table S2), 1.25 U GoTaq® Flexi DNA polymerase (Promega), 1µL of DNA template and filled u with nuclease-free water (NFW). The cycling conditions were an initial denaturation step at 95 °C for 2 min, followed by 35 cycles of denaturation at 95 °C for 1 min, annealing at 51 °C for 1 min and extension at 72 °C for 1 min, followed by a final extension step at 72 °C for 10 min. Positive and negative controls were included in each PCR batch. PCR products were visualised by electrophoresis on a 1.5% agarose gel at 100 V for 85 min. The Leucosphyrus Group produced an expected fragment of ± 870 bp.

Leucosphyrus Group specimens that were positive for the *Plasmodium* genus, together with representative Leucosphyrus Group specimens from each sampling station per Dusun, were selected for Sanger sequencing to confirm *Anopheles* species identity, with a total of up to 30 specimens analysed. The amplification products of single-point ITS2 PCR were purified using ExoSAP-ITTM (Applied Biosystems by Thermo Fisher Scientific, Vilnius, Lithuania) and sequenced using the forward primer and reverse with BigDye(R) Terminator v3.1 Cycle Sequencing Kit (Applied Biosystems by Thermo Fisher Scientific) at the Macrogen sequencing facility (Macrogen Inc., Seoul, Korea). The DNA sequences were trimmed and quality-checked, followed by alignment and consensus sequence assembly. The resulting consensus sequences were then analyzed using BLAST and compared against *An. dirus* reference sequences (KP298432.1; MF326506.1).

1. **Dirus complex species identification PCR (DiCSIP) assay**

The DiCSIP assay identified five members of the Dirus complex, as described by Saeung et al. (2024). The reaction master mix containing 1x GoTaq green buffer (Promega), 2.0 mM of MgCl_2_ (Promega), DMSO 4%, 0.2 mM of dNTP mix (Promega), 0.1 µM of each primer (DiCSIP-Uni-Fwd, DiCSIP-Rev-AC, D-B, D-D and DiCSIP-Rev-F) (Table S2), 1.25 U GoTaq® Flexi DNA polymerase (Promega), 0.5 µL of template and NFW to 12.5 µL. The cycling parameters were an initial denaturation at 94 °C for 2 min, followed by 35 cycles of denaturation at 94 °C for 20 sec, annealing at 62 °C for 20 sec and extension at 72 °C for 1 min, with a final extension step at 72 °C for 5 min. Each PCR batch included appropriate positive (all Dirus Complex species) and negative controls. The amplification products were visualised through an electrophoresis process.

1. **Scanloni-specific PCR (SSP) assay**

The SSP assay, as described by Saeung et al. (2024), was used to differentiate *An. dirus* and *An*. *scanloni* when the PCR products of DiCSIP assay showed band sizes similar to *An. dirus* (521 bp) and *An. scanloni* (528 bp). The 12.5 µL master mix reaction was 1x GoTaq green buffer (Promega), 2.0 mM of MgCl2 (Promega), DMSO 4%, 0.2 mM of dNTP mix (Promega), 0.2 µM of primer DiCSIP-Fwd-C and DiCSIP-Rev-AC (Table S2), 1.25 U GoTaq® Flexi DNA polymerase (Promega), 0.5 µL of gDNA template and topped up with NFW. The cycling conditions were as follows: 94 °C for 2 min; followed by 35 cycles of denaturation at 94 °C for 20 sec, annealing at 62 °C for 20 sec and extension at 72 °C for 1 min, followed by a final extension step at 72 °C for 5 min. Positive (*An. scanloni*) and negative control (NFW template only) controls were included in each PCR batch. The amplified PCR products were separated by 1.5% agarose gel electrophoresis and visualised.

**Table S2.** List of primer sequences and modified annealing temperatures of the Leucosphyrus Group and Dirus Complex

| **References** | **PCR method** | **Gene target** | **Primer** | **Sequence (5′-3′)** | **Annealing temp. (°C)** | **Targeted species** | **DNA size (bp)** |
| --- | --- | --- | --- | --- | --- | --- | --- |
| Beebe and Saul, 1995 | Single-point PCR | ITS2 | ITS2A | TGT GAA CTG CAG GAC ACAT | 51 | Leucsphyrus Group | 870 |
|  |  |  | ITS2B | TAT GCT TAA ATT CAG GGG GT |  |  |  |
| Saeung et al., 2024 | DiCSIP | ITS2 | DiCSIP-Uni-Fwd | GAG TGA TGG ATA CAG AGC GGG | 62 | - | - |
|  |  |  | DiCSIP-Rev-AC | ATC ACT CCA CCT GAC CGG CAA C |  | *An. dirus*  *An. scanloni* | 521-528 |
|  |  |  | D-B | CGG GAT ATG GGT CGG CC |  | *An. cracens* | 435 |
|  |  |  | D-D | GCG CGG GAC CGT CCG TT |  | *An. baimai* | 225 |
|  |  |  | DiCSIP-Rev-F | TCC GCA GCG CAG AGC G |  | *An. nemophilous* | 305 |
|  | SSP |  | DiCSIP-Fwd-C | GCT CCC ACA CAC ACA CAC | 62 | *An. scanloni* | 300 |
|  |  |  | DiCSIP-Rev-AC | ATC ACT CCA CCT GAC CGG CAA C |  |  |  |

**Detection of *Plasmodium* malaria parasites**

1. **Detection of *Plasmodium* spp.**

*Plasmodium* genus infection was detected using highly sensitive reverse transcriptase real-time polymerase chain reaction (RT-qPCR), as described by Kamau et al. (2011) and Braima et al. (2024). The master mix reaction consisted of 1x Taqman-PCR master mix (Applied Biosystems by Thermo Fisher Scientific, Warrington, UK), 0.4 µM of each forward and reverse KamG primers, 0.2 µM of KamGP probe (Table S3), 1 µL of cDNA template and NFW to 10 µL total volume. PCR cycling reactions of duplicate samples, including positive and negative controls, were 2 min at 60°C, 10 min at 95°C, 45 cycles of 15 s at 95°C, and 1 min at 60°C. The RT-qPCR assay was performed using Rotor-Gene Q (Qiagen, Hilden, Germany).

1. **Identification of human malaria parasites**

The cDNA of *Plasmodium* genus positive specimens was analysed for species-specific *Plasmodium* infection by reverse transcriptase nested PCR (RT-nested PCR), using human primers as described by Snounou et al. (1993) and validated by Braima et al. (2024). The nest-1 PCR reaction mixture contained 1x GoTaq green buffer (Promega), 3 mM of MgCl_2_ (Promega), 0.2 mM of dNTP mix (Promega), 0.25 µM of each primer (rPLU1 and rPLU5) (Table S3), 1.25 U GoTaq® Flexi DNA polymerase (Promega), and 4 µL of cDNA template to 50 µL with NFW. The cycling conditions were an initial denaturation for 4 min at 94°C; 35 cycles of 30 sec at 94°C, 1 min at 58°C and 2 min at 72°C; followed by 4 min at 72°C for final extension. The PCR products from nest-1 were the template for nest-2. Nest-1 PCR products were diluted with NFW 1:20. The 20 µL reaction mixture contained 1x GoTaq green buffer (Promega), 3 mM of MgCl_2_ (Promega), 0.2 mM of dNTP mix (Promega), 0.25 µM of each species-specific *Plasmodium* primer (Table S3), 1 U GoTaq® Flexi DNA polymerase (Promega), 2 µL of diluted nest-1 PCR product and NFW. The PCR conditions were an initial denaturation for 4 min at 94°C, 35 cycles of 30 sec at 94°C, 1 min at the primer-specific annealing temperature (Table S3), and 1 min at 72°C, followed by a final extension for 5 min at 72 °C. The amplified PCR products were separated by 1.5% agarose gel electrophoresis and visualised.

1. **Identification of macaque malaria parasites**

A separate RT-nPCR assay was conducted to identify four zoonotic *Plasmodium* species, including *Plasmodium inui*, *Plasmodium cynomolgi*, *Plasmodium coatneyi*, and *Plasmodium fieldi*, using the protocols outlined by Lee et al. (2011) and Braima et al. (2024). In nest-1, the master mix contained 1x GoTaq green buffer (Promega), 3 mM MgCl₂ (Promega), 0.2 mM dNTPs (Promega), 0.25 µM of each rPLU1 and rPLU5 primers (Table S3), 1.25 U of GoTaq® Flexi DNA polymerase (Promega), and 4 µL of cDNA template, with nuclease-free water (NFW) added to reach a final volume of 50 µL. The thermal cycling protocol involved an initial denaturation at 94°C for 4 minutes, followed by 35 cycles of 30 seconds at 94°C, 1 minute at 58°C, and 2 minutes at 72°C, with a final 4-minute extension at 72°C.

In nest-2, the PCR products from nest-1 were diluted 1:10 with NFW and used as a template. The nest-2 master mix included 1x GoTaq green buffer (Promega), 3 mM MgCl₂ (Promega), 0.2 mM dNTPs (Promega), 0.25 µM of species-specific Plasmodium primers (Table S3), 1U GoTaq® Flexi DNA polymerase (Promega), 2 µL of the diluted nest-1 product, and NFW to reach a total volume of 20 µL. The thermal cycling steps started with an initial denaturation at 94°C for 4 minutes, followed by 35 cycles of 30 seconds at 94°C, a 1-minute annealing phase (primer-specific; see Table S3), and 1 minute at 72°C, with a final extension of 5 minutes at 72°C.

1. **Detection of *Plasmodium knowlesi***

For identifying *P. knowlesi*, a RT-hemi nPCR was followed, as described by Imwong et al. (2009) and Braima et al. (2024). The master mix for the nest-1 consisted of 1x GoTaq green buffer (Promega), 2.5 mM of MgCl_2_ (Promega), 0.5 mM of dNTP mix (Promega), 0.25 µM of each PKF1160 and PKR1150 primers (Table S3), 1.25 U GoTaq® Flexi DNA polymerase (Promega), and 1 µL of cDNA template to 25 µL with NFW. The cycling conditions started with an initial denaturation for 5 min at 95°C; 35 cycles of 1 min at 94°C, 1 min at 55°C and 1 min at 72°C; followed by 5 min at 72°C for final extension. Twenty µL of nest 2 master mix reaction contained 1x GoTaq green buffer (Promega), 3 mM of MgCl_2_ (Promega), 0.5 mM of dNTP mix (Promega), 0.25 µM of each primer PKF1140 and PKR1150 primers, 1 U GoTaq® Flexi DNA polymerase (Promega), 1 µL of 1-nest PCR product and NFW. The thermal conditions were an initial denaturation for 5 min at 95°C; 35 cycles of 1 min at 94°C, 1 min at 50°C and 1 min at 72°C; followed by 5 min at 72°C for final extension. The amplified PCR products were separated by 1.5% agarose gel electrophoresis and visualised.

**Table S3.** List of primer sequences and modified annealing temperatures of *Plasmodium* genus and species-specific PCR assays

| **References** | **PCR method** | **Gene target** | **Primer/ Probe** | **Sequence (5′-3′)** | **Annealing temp. (°C)** | **Targeted species** | **DNA size (bp)** |
| --- | --- | --- | --- | --- | --- | --- | --- |
| Kamau et al., 2011 | RT-qPCR | 18S rRNA | KamGF | GCT CTT TCT TGA TTT CTT GGA | 60 | *Plasmodium* spp. | - |
|  |  |  | KamGR | AGC AGG TTA AGA TCT CGT TCG |  |  |  |
|  |  |  | KamGP | FAM-ATG GCC GTT TTT AGT TCG TG-BHQ1 |  |  |  |
| Snounou et al., 1993 | Nested PCR | 18S rRNA | rPLU 1 | TCA AAG ATT AAG CCA TGC AAG TGA | 58 | - | - |
|  |  |  | rPLU 5 | CCT GTT GTT GCC TTA AAC TCC |  |  |  |
|  |  |  | rFAL1 | TTA AAC TGG TTT GGG AAA ACC AAA TAT ATT | 58 | *P. falciparum* | 205 |
|  |  |  | rFAL2 | ACA CAA TGA ACT CAA TCA TGA CTA CCC GTC |  |  |  |
|  |  |  | rVIV1 | CGA CTT CCA AGC CGA AGC AAA GAA AG | 58 | *P. vivax* | 120 |
|  |  |  | rVIV2 | TCC TTA CTT CTA GCT TAA TCC ACA TAA CTG ATA C |  |  |  |
|  |  |  | rOVA1 | ATC TCT TTT GCT ATT TTT TAG TAT TGG AGA | 58 | *P. ovale* | 880 |
|  |  |  | rOVA2 | GGA AAA GGA CAC ATT ATT CIG TAT CCT AGT G |  |  |  |
|  |  |  | rMAL1 | ATA ACA TAG TTG TAC GTT AAG AAT AAC CGC | 58 | *P. malariae* | 144 |
|  |  |  | rMAL2 | AAA ATT CCC ATG CAT AAA AAA TTA TAC AAA |  |  |  |
| Lee et al., 2011 | Nested PCR | 18S rRNA | rPLU 1 | TCA AAG ATT AAG CCA TGC AAG TGA | 58 | *-* | - |
|  |  |  | rPLU 5 | CCT GTT GTT GCC TTA AAC TCC |  |  |  |
|  |  |  | PctF1 | CGC TTT TAG CTT AAA TCC ACA TAA CAG AC | 60 | *P. coatneyi* | 503 |
|  |  |  | PctR1 | GAG TCC TAA CCC CGA AGG GAA AGG |  |  |  |
|  |  |  | CY2F | GAT TTG CTA AAT TGC GGT CG | 60 | *P. cynomolgi* | 137 |
|  |  |  | CY4R | CGG TAT GAT AAG CCA GGG AAG T |  |  |  |
|  |  |  | PfldF1 | GGT CT TTT TTT TGC TTC GGT AAT TA | 63 | *P. fieldi* | 421 |
|  |  |  | PfldR2 | AGG CAC TGA AGG AAG CAA TCTA AGA GTT TC |  |  |  |
|  |  |  | PinF2 | CGT ATC GAC TTT GTG GCA TTT TTC TAC | 58 | *P. inui* | 479 |
|  |  |  | INAR3 | GCA ATC TAA GAG TTT TAA CTC CTC |  |  |  |
| Imwong et al., 2009 | Hemi-nested PCR | 18S rRNA | PKF1160 | GAT GCC TCC GCG TAT CGA C | 55 | *-* | - |
|  |  |  | PKR1150 | GAG TTC TAA TCT CCG GAG AGA AAA GA |  |  |  |
|  |  |  | Pkf1140 | GAT TCA TCT ATT AAA AAT TTG CTT C | 50 | *P. knowlesi* | 410 |
|  |  |  | Pkr1150 | GAG TTC TAA TCT CCG GAG AGA AAA GA |  |  |  |

1. **Single-point 18S rRNA PCR for Sanger sequencing**

At least 25 µL of the nested PCR amplification product from *Plasmodium*-positive specimens was purified using ExoSAP-IT™ (Applied Biosystems by Thermo Fisher Scientific, Vilnius, Lithuania) and sequenced in both forward and reverse directions with the BigDye® Terminator v3.1 Cycle Sequencing Kit (Applied Biosystems by Thermo Fisher Scientific) at the Macrogen sequencing facility (Macrogen Inc., Seoul, Korea). The raw DNA sequences were quality-checked, trimmed, aligned, and assembled into consensus sequences. These were then subjected to BLAST analysis and compared with reference sequences of *Plasmodium* species, including *P. vivax* (KT991312.1; KT991270.1), *P. inui* (HM032051.1; FJ619104.1; KU708872.1), *P. coatneyi* (XR_002198261.1), *P. fieldi* (FJ619101.1), and *P. knowlesi* (KT852897.1; OR139098.1).
